# Impact of β-glycerophosphate on the bioenergetic profile of vascular smooth muscle cells

**DOI:** 10.1007/s00109-020-01925-8

**Published:** 2020-06-02

**Authors:** Ioana Alesutan, Franco Moritz, Tatjana Haider, Sun Shouxuan, Can Gollmann-Tepeköylü, Johannes Holfeld, Burkert Pieske, Florian Lang, Kai-Uwe Eckardt, Silke Sophie Heinzmann, Jakob Voelkl

**Affiliations:** 1 Institute for Physiology and Pathophysiology, Johannes Kepler University, Altenberger Strasse 69, 4040 Linz, Austria; 2 DZHK (German Centre for Cardiovascular Research), partner site Berlin, Berlin, Germany; 3 Berlin Institute of Health (BIH), Berlin, Germany; 4 Department of Internal Medicine and Cardiology, Charité – Universitätsmedizin Berlin, Campus Virchow-Klinikum, Berlin, Germany; 5 Analytical BioGeoChemistry, Helmholtz Zentrum München, German Research Center for Environmental Health, Neuherberg, Germany; 6 University Clinic of Cardiac Surgery, Medical University of Innsbruck, Innsbruck, Austria; 7 Department of Internal Medicine and Cardiology, German Heart Center Berlin (DHZB), Berlin, Germany; 8 Department of Physiology I, Eberhard-Karls University, Tubingen, Germany; 9 Department of Nephrology and Medical Intensive Care, Charité – Universitätsmedizin Berlin, Berlin, Germany

**Keywords:** Bioenergetics, Glycolysis, Mitochondrial respiration, β-Glycerophosphate, Vascular calcification, Vascular smooth muscle cells

## Abstract

**Abstract:**

In chronic kidney disease, hyperphosphatemia is a key pathological factor promoting medial vascular calcification, a common complication associated with cardiovascular events and mortality. This active pathophysiological process involves osteo-/chondrogenic transdifferentiation of vascular smooth muscle cells (VSMCs) via complex intracellular mechanisms that are still incompletely understood. Little is known about the effects of phosphate on the bioenergetic profile of VSMCs during the onset of this process. Therefore, the present study explored the effects of the phosphate donor β-glycerophosphate on cellular bioenergetics of VSMCs. Mitochondrial and glycolytic functions were determined utilizing extracellular flux analysis in primary human aortic VSMCs following exposure to β-glycerophosphate. In VSMCs, β-glycerophosphate increased basal respiration, mitochondrial ATP production as well as proton leak and decreased spare respiratory capacity and coupling efficiency, but did not modify non-mitochondrial or maximal respiration. β-Glycerophosphate-treated VSMCs had higher ability to increase mitochondrial glutamine and long-chain fatty acid usage as oxidation substrates to meet their energy demand. β-Glycerophosphate did not modify glycolytic function or basal and glycolytic proton efflux rate. In contrast, β-glycerophosphate increased non-glycolytic acidification. β-Glycerophosphate-treated VSMCs had a more oxidative and less glycolytic phenotype, but a reduced ability to respond to stressed conditions via mitochondrial respiration. Moreover, compounds targeting components of mitochondrial respiration modulated β-glycerophosphate-induced oxidative stress, osteo-/chondrogenic signalling and mineralization of VSMCs. In conclusion, β-glycerophosphate modifies key parameters of mitochondrial function and cellular bioenergetics in VSMCs that may contribute to the onset of phenotypical transdifferentiation and calcification. These observations advance the understanding of the role of energy metabolism in VSMC physiology and pathophysiology of vascular calcification during hyperphosphatemia.

**Key messages:**

- β-Glycerophosphate modifies key parameters of mitochondrial respiration in VSMCs.
- β-Glycerophosphate induces changes in mitochondrial fuel choice in VSMCs.
- β-Glycerophosphate promotes a more oxidative and less glycolytic phenotype of VSMCs.
- β-Glycerophosphate triggers mitochondrial-dependent oxidative stress in VSMCs.
- Bioenergetics impact β-glycerophosphate-induced VSMC calcification.

**Electronic supplementary material:**

The online version of this article (10.1007/s00109-020-01925-8) contains supplementary material, which is available to authorized users.

## Introduction

Hyperphosphatemia develops in chronic kidney disease (CKD) patients as a consequence of impaired phosphate excretion by the diseased kidneys [1, 2]. Elevated phosphate levels are considered a decisive cardiovascular risk factor in CKD [3] and predictor of cardiovascular and all-cause mortality [4, 5]. Hyperphosphatemia promotes medial vascular calcification [5, 6], a common complication in CKD [7] considered responsible, at least in part, for the high cardiovascular mortality of these patients [6]. However, no effective treatment options to reduce the progression of vascular calcification in patients with CKD are available so far.

Vascular calcification is an active pathophysiological process promoted mainly by vascular smooth muscle cells (VSMCs) [5, 8]. Phosphate induces transdifferentiation of contractile VSMCs into osteo-/chondroblast-like VSMCs [5], which produce a local pro-calcifying environment that facilitates calcium phosphate precipitation and vascular tissue mineralization [5, 9, 10]. Osteo-/chondrogenic transdifferentiation of VSMCs is regulated by a complex intracellular signalling network, which is still incompletely understood [11]. Identification of the key signalling pathways controlling osteo-/chondrogenic transdifferentiation of VSMCs may provide therapeutic targets for interference with the development of vascular calcification in CKD [5, 9, 12–16].

Recently, VSMC energy metabolism has been identified as part of the mechanisms controlling cell phenotype switch [17–19], including osteo-/chondrogenic transdifferentiation [20–23]. It was suggested that VSMCs may adjust to the new energy demand during transdifferentiation [24] by regulation of cellular metabolic pathways and subsequent ATP production through mitochondrial oxidative phosphorylation and glycolysis [17, 18, 20, 24, 25], the two major cellular energy-producing pathways [19, 26]. During oxidative phosphorylation, electrons are removed from fuels such as glucose, amino acids or fatty acids and passaged through the mitochondrial electron transport chain, leading to reduction of oxygen to water and generation of a proton gradient across the inner mitochondrial membrane [27, 28], which is the driving force for mitochondrial ATP production via the ATP synthase [28]. In turn, glycolysis generates ATP by conversion of glucose to pyruvate [26]. Accumulating evidence has linked metabolic changes related to mitochondrial respiratory function [20–23, 29] and glycolysis [30] with the signalling regulating osteo-/chondrogenic transdifferentiation of VSMCs and vascular calcification. Along those lines, previous studies already suggested that elevated phosphate levels promote mitochondrial dysfunction in VSMCs [21–23, 29] followed by oxidative stress [20, 29, 31], endoplasmic reticulum stress [32] and apoptosis [20], which mediate, at least partly, phosphate-induced vascular calcification [5]. More importantly, restoration of mitochondrial function [21–23] or inhibition of mitochondria-derived oxidative stress [29, 31] was found to inhibit phosphate-induced osteo-/chondrogenic transdifferentiation and calcification of VSMCs. However, the impact of phosphate on the bioenergetics of VSMCs during vascular calcification is still elusive. Especially, details regarding the function of the bioenergetic components in VSMCs during hyperphosphatemia are largely unknown.

Therefore, the present study explored the effects of exposure to the phosphate donor, β-glycerophosphate, on parameters of mitochondrial respiration and glycolytic function as well as on the bioenergetic phenotype of VSMCs. In addition, the effects of metabolic modulators on β-glycerophosphate-induced oxidative stress, osteo-/chondrogenic transdifferentiation and calcification of VSMCs were investigated.

## Materials and methods

A detailed description of the methods is found in Supplementary information.

## Results

To investigate the early effects of phosphate exposure on the bioenergetics of VSMCs, experiments were performed in primary human aortic smooth muscle cells (HAoSMCs) treated with the phosphate donor, β-glycerophosphate [12, 14], for 24 h followed by extracellular flux analysis using the Seahorse technology to analyse parameters of mitochondrial respiration as well as glycolysis by simultaneous time course measurement of the oxygen consumption rate (OCR) and extracellular acidification rate (ECAR), respectively.

**Fig. 1:**
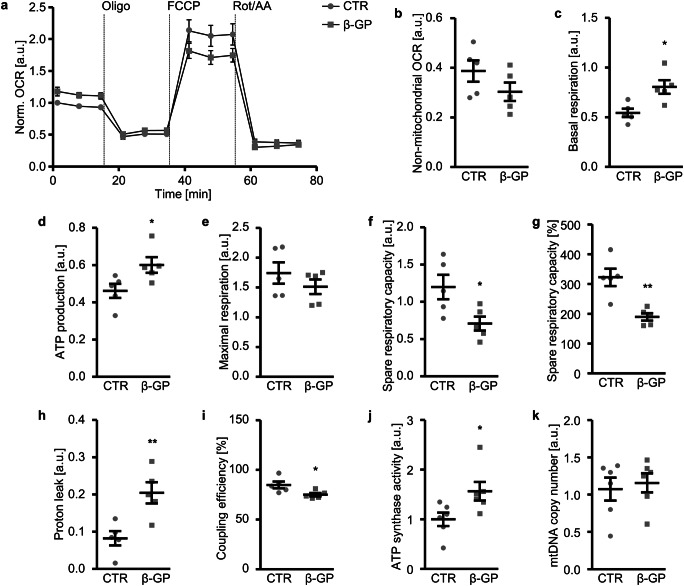
Effect of β-glycerophosphate on mitochondrial function in primary human aortic smooth muscle cells. **a** Arithmetic means ± SEM (*n* = 5; arbitrary units, a.u.) of normalized oxygen consumption rate (OCR) measured over time in HAoSMCs following treatment for 24 h with control (CTR) or β-glycerophosphate (β-GP). Oligomycin (Oligo), FCCP and a mix of rotenone and antimycin A (Rot/AA) were sequentially injected at the indicated times. **b**–**i** Scatter dot plots and arithmetic means ± SEM (*n* = 5) of normalized non-mitochondrial OCR (**b**, a.u.), basal respiration (**c**, a.u.), ATP production (**d**, a.u.), maximal respiration (**e**, a.u.), spare respiratory capacity (**f**, a.u.), spare respiratory capacity (**g**, %), proton leak (**h**, a.u.) and coupling efficiency (**i**, %) in HAoSMCs following treatment for 24 h with control (CTR) or β-glycerophosphate (β-GP). **j** Scatter dot plots and arithmetic means ± SEM (*n* = 6, a.u.) of normalized ATP synthase activity in HAoSMCs following treatment for 24 h with control (CTR) or β-glycerophosphate (β-GP). **k** Scatter dot plots and arithmetic means ± SEM (*n* = 6, a.u.) of normalized mitochondrial DNA (mtDNA) copy number relative to nuclear DNA copy number in HAoSMCs following treatment for 24 h with control (CTR) or β-glycerophosphate (β-GP). *(*p* < 0.05), **(*p* < 0.01) significant vs. control HAoSMCs

The effects of β-glycerophosphate on key parameters of mitochondrial function were analysed by monitoring the OCR during sequential addition of compounds targeting specific components of the mitochondrial electron transport chain: oligomycin that inhibits ATP synthase (complex V) and FCCP that collapses the proton gradient and disrupts the mitochondrial membrane potential, leading to a maximal oxygen consumption by complex IV and a mix of rotenone and antimycin A that inhibit complex I and complex III, respectively, and, thus, block mitochondrial respiration (Fig. 1a). As a result, non-mitochondrial respiration, attributed to other cellular enzymes that consume oxygen, was similar in control-treated and β-glycerophosphate-treated HAoSMCs (Fig. 1b). β-Glycerophosphate-treated HAoSMCs had a significantly higher baseline respiration than control HAoSMCs (Fig. 1c), suggesting an increased energy demand under hyperphosphatemic conditions. In accordance, mitochondrial ATP production was increased in HAoSMCs exposed to β-glycerophosphate (Fig. 1d), to meet the high energetic needs. The maximum rate of respiration was not significantly different between the groups (Fig. 1e), and the spare respiratory capacity was significantly lower in β-glycerophosphate-treated than control-treated HAoSMCs, and, thus, the respiration was closer to their maximum capability (Fig. 1f, g). Furthermore, the proton leak, the baseline respiration not linked to mitochondrial ATP production, was significantly increased by β-glycerophosphate exposure, an observation pointing towards increased mitochondrial damage (Fig. 1h). Accordingly, the coupling efficiency of baseline respiration to mitochondrial ATP production was significantly reduced in β-glycerophosphate-treated as compared to control HAoSMCs (Fig. 1i). β-Glycerophosphate significantly increased ATP synthase activity in HAoSMCs (Fig. 1j). The key parameters of mitochondrial function were modified following β-glycerophosphate treatment without significant effects on mitochondrial DNA copy number (Fig. 1k).

**Fig. 2:**
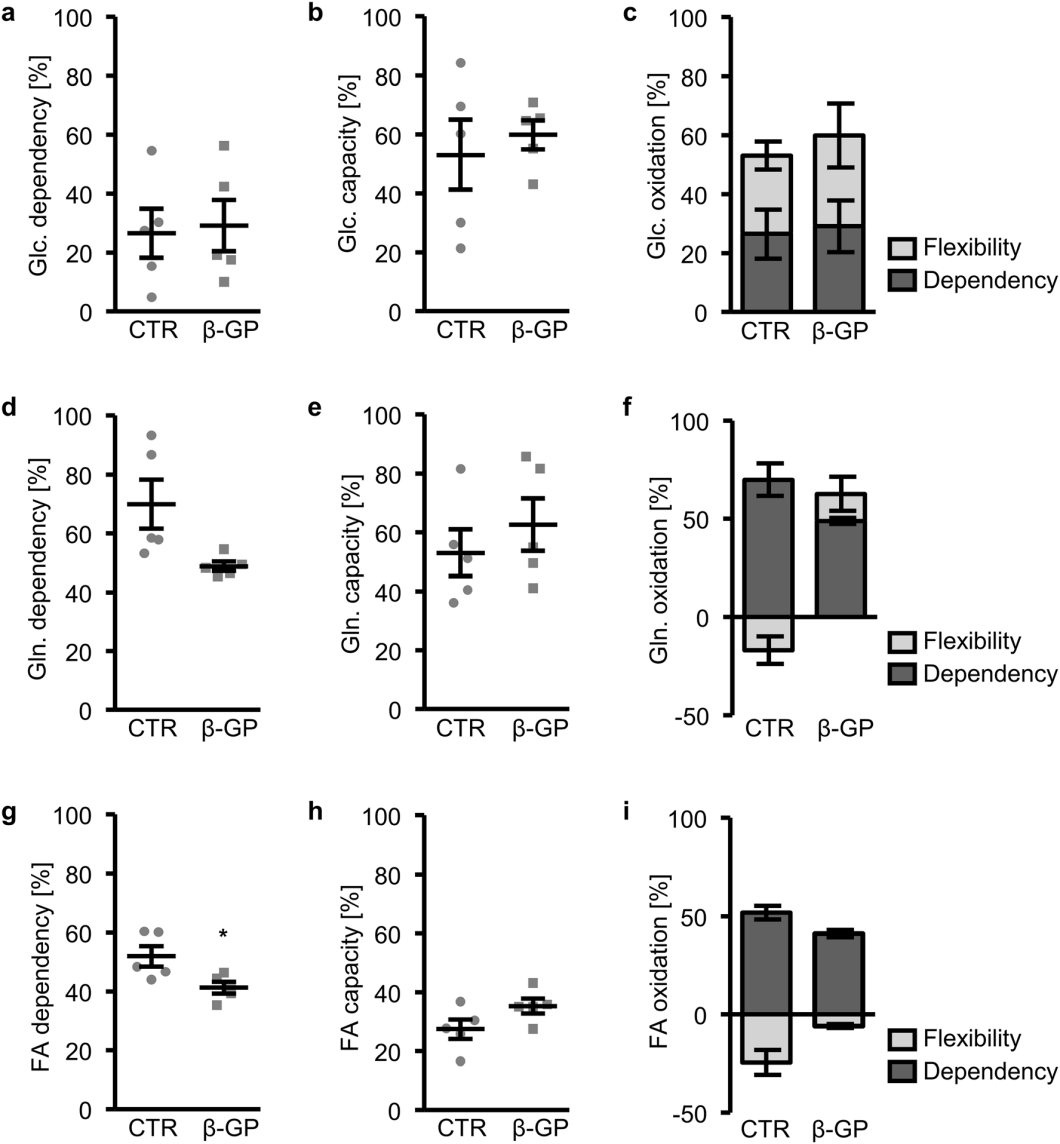
Effect of β-glycerophosphate on mitochondrial fuel usage in primary human aortic smooth muscle cells. **a**, **b** Scatter dot plots and arithmetic means ± SEM (*n* = 5) of glucose (Glc.) oxidation pathway dependency (**a**) and capacity (**b**) (% from glucose + glutamine + long-chain fatty acid oxidation) in HAoSMCs following treatment for 24 h with control (CTR) or β-glycerophosphate (β-GP). **c** Arithmetic means ± SEM (*n* = 5) of glucose oxidation (% from glucose + glutamine + long-chain fatty acid oxidation) in HAoSMCs following treatment for 24 h with control (CTR) or β-glycerophosphate (β-GP). **d**, **e** Scatter dot plots and arithmetic means ± SEM (*n* = 5) of glutamine (Gln.) oxidation pathway dependency (**d**) and capacity (**e**) (% from glucose + glutamine + long-chain fatty acid oxidation) in HAoSMCs following treatment for 24 h with control (CTR) or β-glycerophosphate (β-GP). **f** Arithmetic means ± SEM (*n* = 5) of glutamine oxidation (% from glucose + glutamine + long-chain fatty acid oxidation) in HAoSMCs following treatment for 24 h with control (CTR) or β-glycerophosphate (β-GP). **g**, **h** Scatter dot plots and arithmetic means ± SEM (*n* = 5) of fatty acid (FA) oxidation pathway dependency (**g**) and capacity (**h**) (% from glucose + glutamine + long-chain fatty acid oxidation) in HAoSMCs following treatment for 24 h with control (CTR) or β-glycerophosphate (β-GP). **i** Arithmetic means ± SEM (*n* = 5) of fatty acid oxidation (% from glucose + glutamine + long-chain fatty acid oxidation) in HAoSMCs following treatment for 24 h with control (CTR) or β-glycerophosphate (β-GP). *(*p* < 0.05) significant vs. control HAoSMCs

To explore whether β-glycerophosphate-treated HAoSMCs might adjust their mitochondrial fuel usage to meet their high energy demand, OCR was monitored in the presence or absence of UK5099, BPTES and/or Etomoxir, inhibitors of glucose, glutamine and long-chain fatty acid oxidation pathways, respectively (Suppl. Figs.1–3). Inhibition of one oxidation pathway before or after simultaneous inhibition of the other two alternative pathways allows for determination of dependency, capacity and flexibility to oxidize a specific mitochondrial fuel. As shown in Fig. 2a–c, mitochondrial glucose oxidation was not significantly affected by β-glycerophosphate exposure. In contrast, the glutamine dependency, showing the reliance on glutamine oxidation to maintain their baseline respiration, tended to be lower in β-glycerophosphate-treated than in control-treated HAoSMCs, a difference, however, not reaching statistical significance (*p* = 0.064; Fig. 2d). The capacity to use glutamine oxidation was not significantly different between the groups (Fig. 2e), but the glutamine flexibility and, thus, the ability of HAoSMCs to increase glutamine oxidation to compensate for the other two mitochondrial fuels was significantly increased following prolonged exposure to β-glycerophosphate (*p* = 0.0258; Fig. 2f). Similarly, for the long-chain fatty acid oxidation pathway, the dependency was significantly lower (Fig. 2g), while the capacity tended to be higher (*p* = 0.0944; Fig. 2h) and, thus, the flexibility was significantly increased (*p* = 0.042; Fig. 2i) in β-glycerophosphate-treated HAoSMCs than control HAoSMCs. Thus, β-glycerophosphate-treated HAoSMCs had a higher ability to increase mitochondrial glutamine and long-chain fatty acid usage as substrates for oxidation to meet their energy demand.

**Fig. 3:**
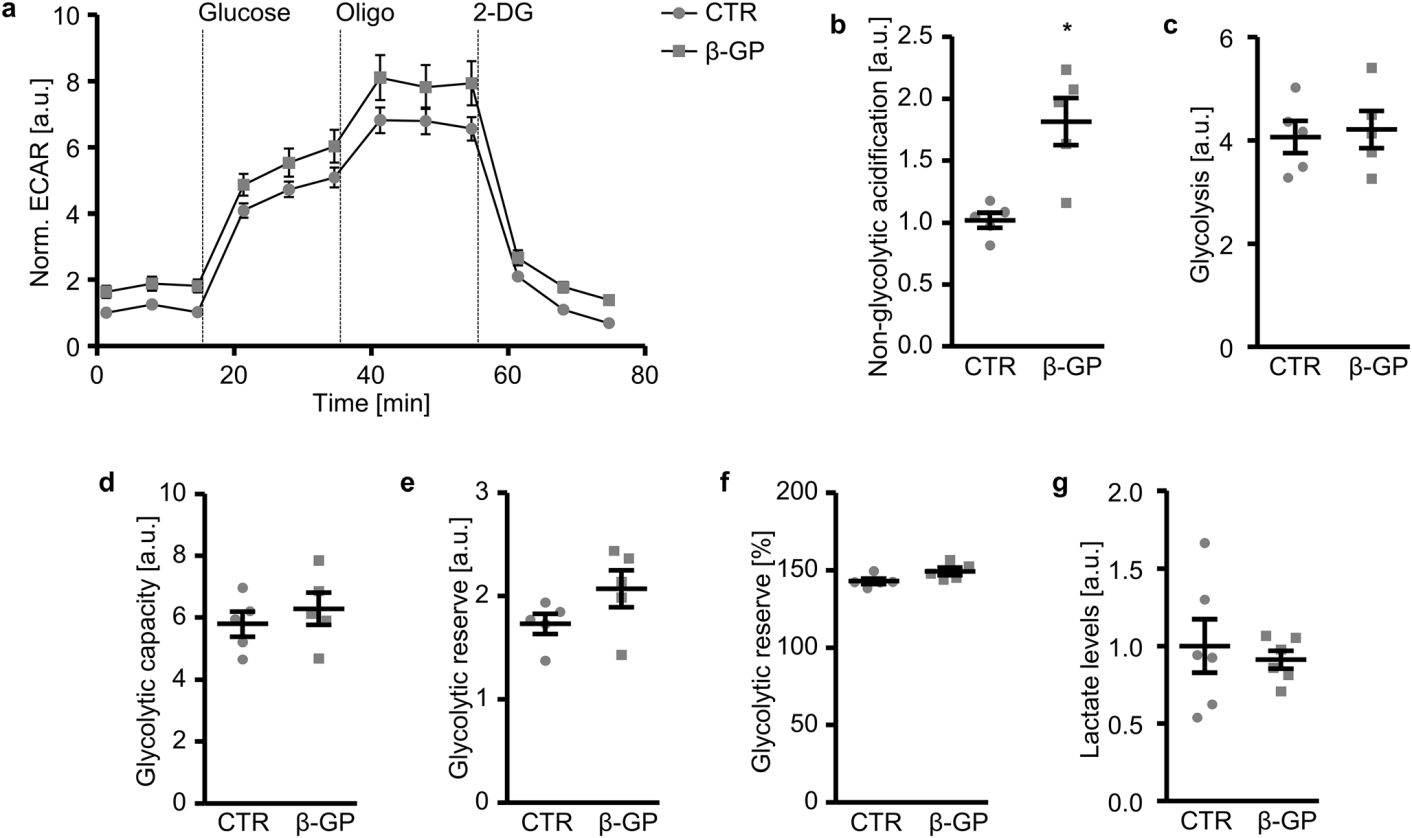
Effect of β-glycerophosphate on glycolytic function in primary human aortic smooth muscle cells. **a** Arithmetic means ± SEM (*n* = 5; arbitrary units, a.u.) of normalized extracellular acidification rate (ECAR) measured over time in HAoSMCs following treatment for 24 h with control (CTR) or β-glycerophosphate (β-GP). Glucose, oligomycin (Oligo) and 2-DG were sequentially injected at the indicated times. **b**–**f** Scatter dot plots and arithmetic means ± SEM (*n* = 5) of normalized non-glycolytic acidification (**b**, a.u.), glycolysis (**c**, a.u.), glycolytic capacity (**d**, a.u.), glycolytic reserve (**e**, a.u.) and glycolytic reserve (**f**, %) in HAoSMCs following treatment for 24 h with control (CTR) or β-glycerophosphate (β-GP). **g** Scatter dot plots and arithmetic means ± SEM (*n* = 6, a.u.) of normalized lactate levels in cell culture medium of HAoSMCs following treatment for 24 h with control (CTR) or β-glycerophosphate (β-GP). *(*p* < 0.05) significant vs. control HAoSMCs

The effects of β-glycerophosphate on key parameters of glycolytic function were analysed by monitoring the ECAR (Fig. 3a). ECAR was measured in the absence of glucose and pyruvate, and, thus, the extracellular acidification, driven by other cellular processes than glycolysis, was significantly higher in β-glycerophosphate-treated than in control-treated HAoSMCs (Fig. 3b). Addition of glucose induced an increase in ECAR to similar levels, reflecting a similar glycolysis in both groups at baseline conditions (Fig. 3c). Blocking the mitochondrial ATP production with oligomycin to induce the shift of cellular ATP production towards glycolysis allowed for determination of the maximum glycolytic capacity, which was also not significantly changed following β-glycerophosphate treatment (Fig. 3d). However, the glycolytic reserve, showing the ability to respond through glycolysis to an increased energy requirement, tended to be higher in β-glycerophosphate-treated HAoSMCs (*p* = 0.133 for Fig. 3e; *p* = 0.067 for Fig. 3f). Lactate levels released in the cell culture medium were not significantly changed by β-glycerophosphate (Fig. 3g).

**Fig. 4:**
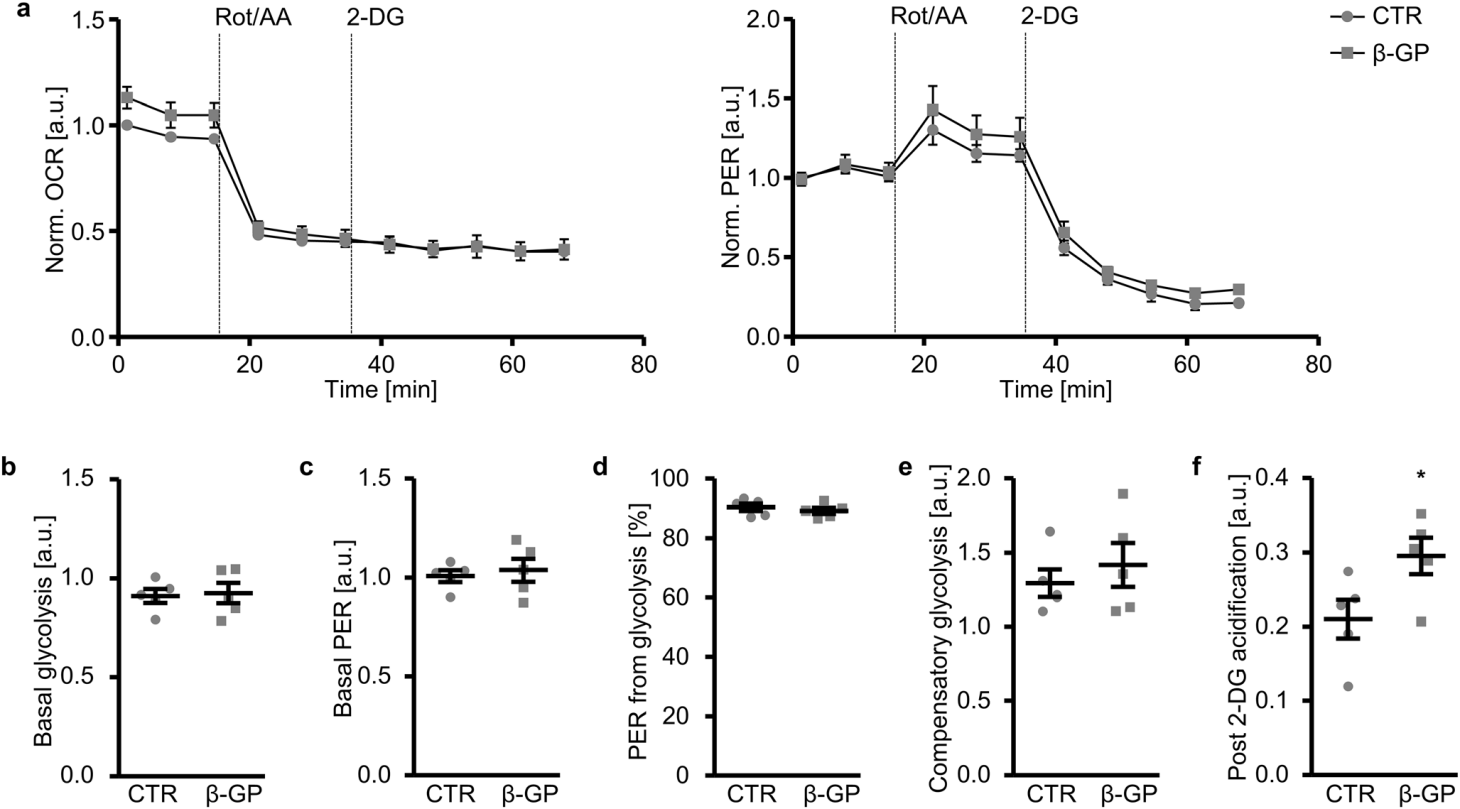
Effect of β-glycerophosphate on glycolytic rates in primary human aortic smooth muscle cells. **a** Arithmetic means ± SEM (*n* = 5; arbitrary units, a.u.) of normalized oxygen consumption rate (OCR) and proton efflux rate (PER) measured over time in HAoSMCs following treatment for 24 h with control (CTR) or β-glycerophosphate (β-GP). A mix of rotenone and antimycin A (Rot/AA) and 2-DG were sequentially injected at the indicated times. **b**–**f**. Scatter dot plots and arithmetic means ± SEM (*n* = 5) of normalized basal glycolysis (**b**, a.u.), basal PER (**c**, a.u.), PER from glycolysis (**d**, %), compensatory glycolysis (**e**, a.u.) and post 2-DG acidification (**f**, a.u.) in HAoSMCs following treatment for 24 h with control (CTR) or β-glycerophosphate (β-GP). *(*p* < 0.05) significant vs. control HAoSMCs

The effects on glycolytic rates during baseline conditions and on compensatory glycolysis after inhibition of mitochondrial oxidative phosphorylation with rotenone and antimycin A were analysed by simultaneous monitoring of the OCR and ECAR and the subsequent conversion of data into proton efflux rates (PER) (Fig. 4a). β-Glycerophosphate-treated HAoSMCs had similar baseline glycolysis (Fig. 4b), baseline total PER (Fig. 4c), baseline PER from glycolysis (Fig. 4d) as well as compensatory glycolysis (Fig. 4e) as control-treated HAoSMCs. The extracellular acidification resulting from other cellular processes than mitochondrial respiration and glycolysis, measured in the presence of rotenone and antimycin A and of 2-DG (2-deoxy-glucose), was significantly higher in β-glycerophosphate-treated HAoSMCs (Fig. 4f). Taken together, prolonged exposure to β-glycerophosphate did not modify the glycolytic function in HAoSMCs, but stimulated other cellular processes which promote extracellular acidification.

**Fig. 5:**
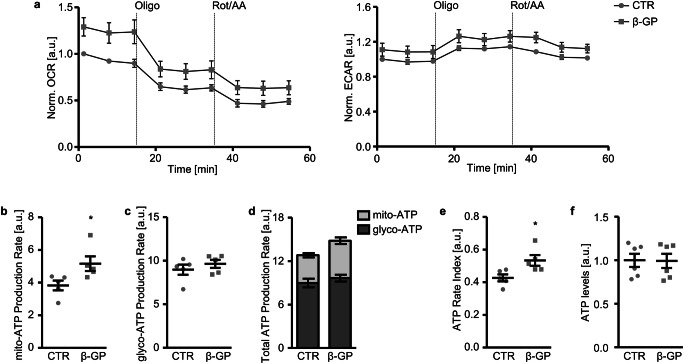
Effect of β-glycerophosphate on the ATP production rates in primary human aortic smooth muscle cells. **a** Arithmetic means ± SEM (*n* = 5; arbitrary units, a.u.) of normalized oxygen consumption rate (OCR) and extracellular acidification rate (ECAR) measured over time in HAoSMCs following treatment for 24 h with control (CTR) or β-glycerophosphate (β-GP). Oligomycin (Oligo) and a mix of rotenone and antimycin A (Rot/AA) were sequentially injected at the indicated times. **b**, **c** Scatter dot plots and arithmetic means ± SEM (*n* = 5, a.u.) of normalized mitochondrial (**b**, mito-ATP) and glycolytic (**c**, glyco-ATP) ATP production rates in HAoSMCs following treatment for 24 h with control (CTR) or β-glycerophosphate (β-GP). **d** Arithmetic means ± SEM (*n* = 5, a.u.) of normalized total ATP production rates in HAoSMCs following treatment for 24 h with control (CTR) or β-glycerophosphate (β-GP). **e** Scatter dot plots and arithmetic means ± SEM (*n* = 5, a.u.) of ATP rate index in HAoSMCs following treatment for 24 h with control (CTR) or β-glycerophosphate (β-GP). **f** Scatter dot plots and arithmetic means ± SEM (*n* = 6, a.u.) of normalized ATP levels in HAoSMCs following treatment for 24 h with control (CTR) or β-glycerophosphate (β-GP). *(*p* < 0.05) significant vs. control HAoSMCs

To further investigate the effects of β-glycerophosphate on the bioenergetics in HAoSMCs, the rates of ATP production from mitochondrial oxidative phosphorylation and glycolysis in response to the increased ATP demand were determined (Fig. 5a). In control HAoSMCs, 29.89% of ATP production resulted from mitochondrial oxidative phosphorylation and 70.11% from glycolysis. As shown in Fig. 5b, the rate of ATP production from oxidative phosphorylation was significantly higher in β-glycerophosphate-treated than in control HAoSMCs. The rate of ATP production from glycolysis (Fig. 5c) and total ATP production rate (Fig. 5d) were not significantly modified by β-glycerophosphate. The ATP production from mitochondrial oxidative phosphorylation increased to 34.84% of total ATP production rate in β-glycerophosphate-treated HAoSMCs. Moreover, the ATP rate index, the ratio of mitochondrial ATP production rate and glycolytic ATP production rate, was significantly increased by β-glycerophosphate, reflecting a more oxidative and less glycolytic phenotype of HAoSMCs exposed to high β-glycerophosphate concentrations (Fig. 5e). Total cellular ATP levels were not significantly different between the groups (Fig. 5f).

**Fig. 6:**
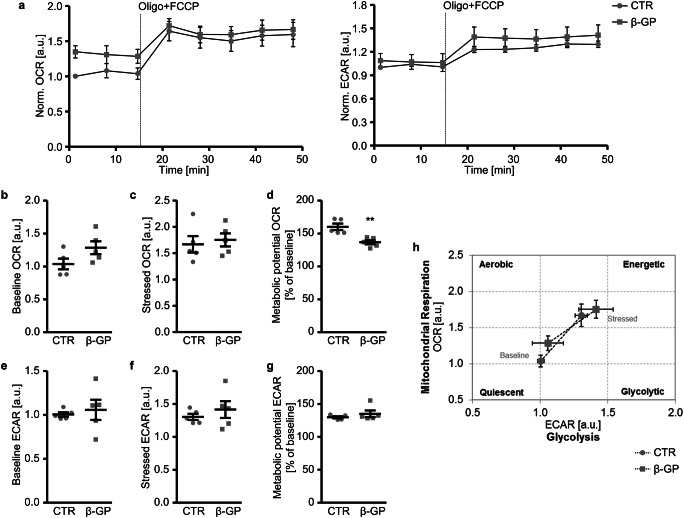
Effect of β-glycerophosphate on cell energy phenotype of primary human aortic smooth muscle cells. **a** Arithmetic means ± SEM (*n* = 5; arbitrary units, a.u.) of normalized oxygen consumption rate (OCR) and extracellular acidification rate (ECAR) measured over time in HAoSMCs following treatment for 24 h with control (CTR) or β-glycerophosphate (β-GP). Oligomycin (Oligo) together with FCCP were injected at the indicated time. **b**–**d** Scatter dot plots and arithmetic means ± SEM (*n* = 5) of normalized baseline OCR (**b**, a.u.), stressed OCR (**c**, a.u.) and metabolic potential for respiration (**d**, %) in HAoSMCs following treatment for 24 h with control (CTR) or β-glycerophosphate (β-GP). **e**–**g** Scatter dot plots and arithmetic means ± SEM (*n* = 5) of normalized baseline ECAR (**e**, a.u.), stressed ECAR (**f**, a.u.) and metabolic potential for glycolysis (**g**, %) in HAoSMCs following treatment for 24 h with control (CTR) or β-glycerophosphate (β-GP). **h** Arithmetic means ± SEM (*n* = 5; a.u.) showing baseline and stressed energy phenotypes of HAoSMCs following treatment for 24 h with control (CTR) or β-glycerophosphate (β-GP). **(*p* < 0.01) significant vs. control HAoSMCs

To explore the effects of β-glycerophosphate on the energetic phenotype under baseline and stressed conditions, a stressed phenotype was determined by addition of oligomycin together with FCCP to induce a high energy demand for HAoSMCs (Fig. 6a). Under baseline conditions, the respiration was increased (*p* = 0.089; Fig. 6b), while the extracellular acidification was not significantly affected (Fig. 6e) by β-glycerophosphate. Addition of stressors leads to increases in OCR (Fig. 6c) and ECAR (Fig. 6f) to similarly high levels in both control- and β-glycerophosphate-treated HAoSMCs. The metabolic potential of HAoSMCs to meet the energy demands induced by stressors was significantly lower for respiration (Fig. 6d) and not modified for glycolysis (Fig. 6g) following β-glycerophosphate exposure, suggesting that the ability of β-glycerophosphate-treated HAoSMCs to respond to the high energy requirements during stressed conditions via mitochondrial respiration was reduced (Fig. 6h).

**Fig. 7:**
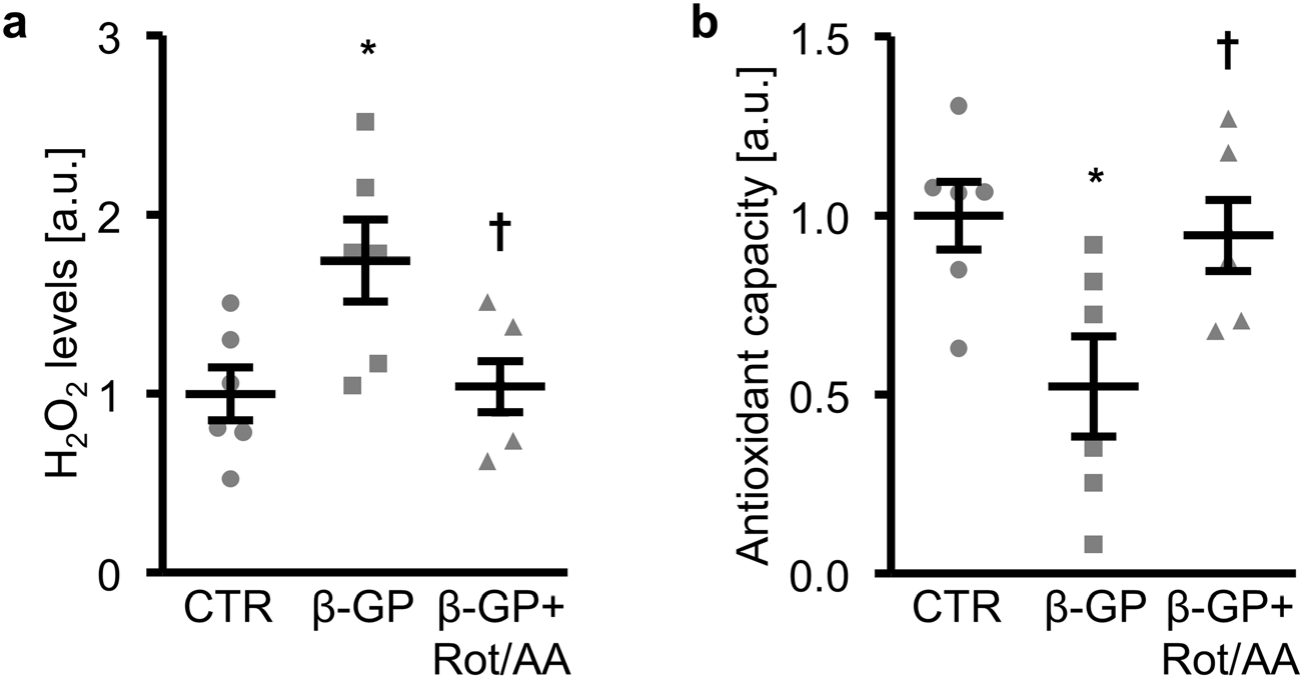
Effect of mitochondrial respiration inhibition on β-glycerophosphate-induced oxidative stress in primary human aortic smooth muscle cells. **a**, **b** Scatter dot plots and arithmetic means ± SEM (*n* = 6; arbitrary units, a.u.) of normalized H_2_O_2_ levels (**a**) and total antioxidant capacity (**b**) in HAoSMCs following treatment for 24 h with control (CTR) or β-glycerophosphate (β-GP) in the presence and absence of a mix of rotenone and antimycin A (Rot/AA). *(*p* < 0.05) significant vs. control HAoSMCs; ^†^(*p* < 0.05) significant vs. HAoSMCs treated with β-GP alone

Next, the role of mitochondrial respiration in β-glycerophosphate-induced oxidative stress was addressed. β-Glycerophosphate promoted oxidative stress in HAoSMCs, as shown by increased H_2_O_2_ levels (Fig. 7a) and reduced total antioxidant capacity (Fig. 7b). These effects were significantly blunted in the presence of a mix of rotenone and antimycin A. Thus, mitochondrial respiration was, at least partly, the source of β-glycerophosphate-induced oxidative stress in HAoSMCs.

**Fig. 8:**
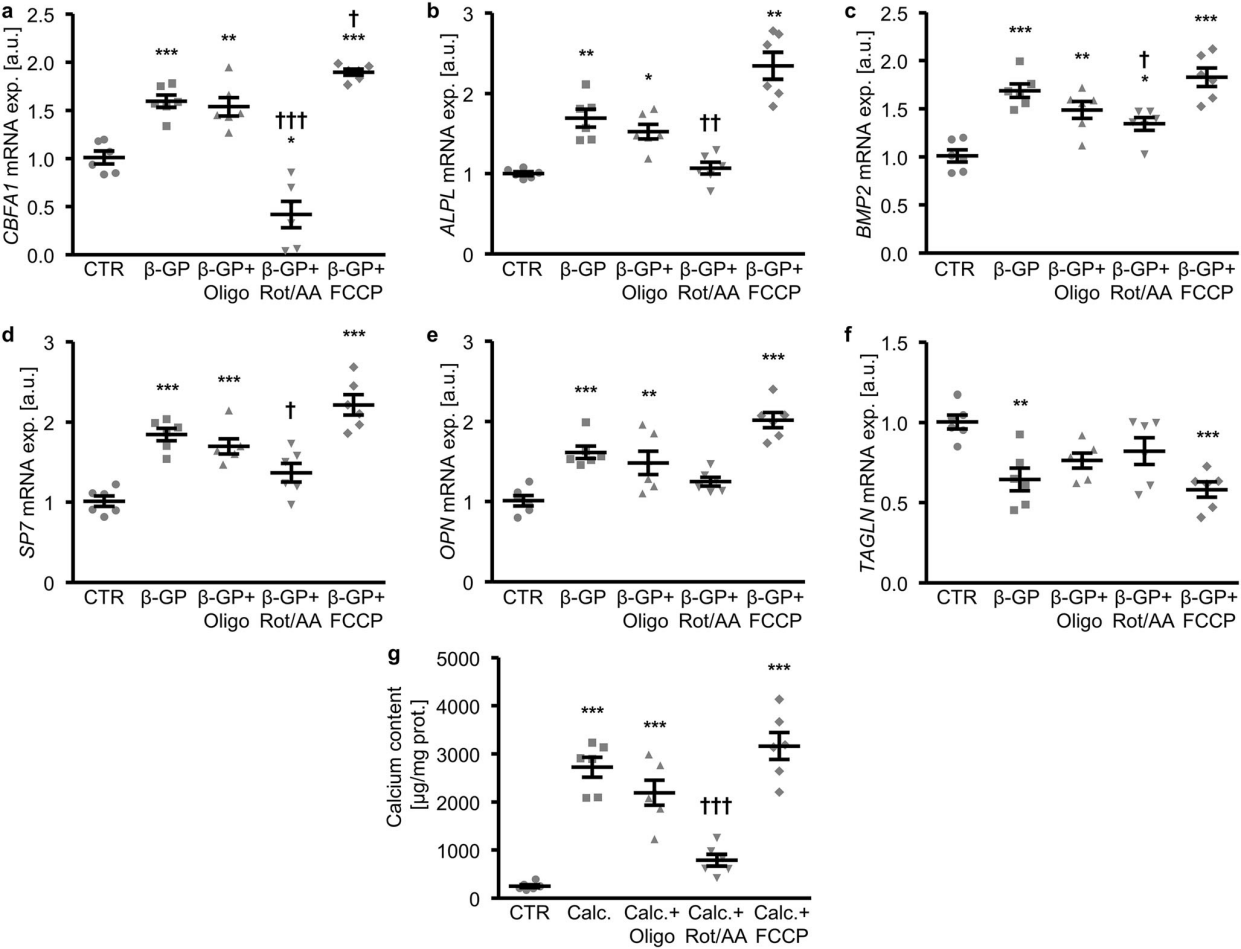
Effect of mitochondrial electron transport chain inhibitors on β-glycerophosphate-induced osteo-/chondrogenic transdifferentiation and calcification of primary human aortic smooth muscle cells. **a**–**f** Scatter dot plots and arithmetic means ± SEM (*n* = 6; arbitrary units, a.u.) of *CBFA1* (**a**), *ALPL* (**b**), *BMP2* (**c**), *SP7* (**d**), *OPN* (**e**) and *TAGLN* (**f**) normalized mRNA expression in HAoSMCs following treatment for 24 h with control (CTR) or β-glycerophosphate (β-GP) in the presence and absence of oligomycin (Oligo), a mix of rotenone and antimycin A (Rot/AA) or FCCP. **g** Scatter dot plots and arithmetic means ± SEM (*n* = 6; μg/mg protein) of calcium content in HAoSMCs following treatment for 11 days with control (CTR) or calcification medium (Calc.; containing β-glycerophosphate and CaCl_2_) in the presence and absence of Oligomycin (Oligo), a mix of rotenone and antimycin A (Rot/AA) or FCCP. *(*p* < 0.05), **(*p* < 0.01), ***(*p* < 0.001) significant vs. control HAoSMCs; ^†^(*p* < 0.05), ^††^(*p* < 0.01), ^†††^(*p* < 0.001) significant vs. HAoSMCs treated with β-GP/Calc. alone

Further, the involvement of cellular bioenergetics in β-glycerophosphate-induced VSMC calcification was investigated. β-Glycerophosphate significantly upregulated osteo-/chondrogenic markers *CBFA1*, *ALPL, BMP2*, *SP7* (gene encoding osterix) and *OPN* mRNA expression in HAoSMCs (Fig. 8a–e). These effects were not significantly changed by oligomycin. A mix of rotenone and antimycin A significantly inhibited β-glycerophosphate-induced *CBFA1*, *ALPL*, *BMP2* and *SP7* and tended to reduce *OPN* mRNA expression (*p* = 0.055). Rotenone and antimycin A reduced *CBFA1* mRNA expression beyond the control levels. Addition of FCCP significantly augmented *CBFA1*, tended to increase *ALPL* (*p* = 0.0631) and *SP7* (*p* = 0.091), but did not significantly affect *BMP2* or *OPN* mRNA expression in β-glycerophosphate-treated HAoSMCs. In addition, *TAGLN* (gene encoding SM22α) mRNA expression was significantly downregulated in HAoSMCs treated with β-glycerophosphate alone or together with FCCP, but not with β-glycerophosphate together with oligomycin or a mix of rotenone and antimycin A (Fig. 8f). Furthermore, the mineralization of HAoSMCs induced by a calcification medium was significantly reduced by additional treatment with a mix of rotenone and antimycin A, but not with oligomycin or FCCP (Fig. 8g). Thus, osteo-/chondrogenic transdifferentiation and calcification of HAoSMCs during high β-glycerophosphate conditions involved, at least partly, changes in mitochondrial function.

## Discussion

The present study provides a detailed analysis of the bioenergetic profile of VSMCs following exposure to high phosphate conditions. The phosphate donor, β-glycerophosphate, not only promotes a more oxidative phenotype with increased basal respiration and mitochondrial ATP production rates as well as changes in mitochondrial fuel choice, but also increases proton leak and, thus, reduces coupling efficiency of baseline respiration to mitochondrial ATP production, pointing towards mitochondrial damage. Accordingly, VSMCs exposed to increased β-glycerophosphate concentrations have a reduced ability to respond to stress through mitochondrial respiration. β-Glycerophosphate does not modify glycolytic flux in VSMCs. Moreover, inhibition of specific components of mitochondrial respiration impacts on β-glycerophosphate-induced osteo-/chondrogenic transdifferentiation and calcification of VSMCs.

ATP, the main energy-storage molecule [33], represents the fuel for a multitude of cellular processes and an intracellular signalling molecule that connects physiological responses with cellular metabolic state [19, 34]. Mitochondrial oxidative phosphorylation and glycolysis are the two major ATP-producing pathways in cells [19, 26]. Mitochondrial respiration has a higher efficiency and represents the preferred pathway for ATP generation in differentiated cells [19, 26, 35, 36]. However, similar to the Warburg effect described in cancer cells [35, 36], VSMCs show high aerobic glycolysis under normal conditions [37, 38], a metabolic characteristic of proliferating cells [26, 36, 39]. In accordance with the previous observations, in primary human aortic VSMCs, glycolysis is responsible on average for approximately 70.1% of total ATP production.

Following exposure to β-glycerophosphate, glycolysis and glycolytic ATP production rates are not modified in VSMCs. However, basal mitochondrial respiration, mitochondrial ATP synthase activity and ATP production rates are increased in β-glycerophosphate-treated VSMCs, leading to a switch in the bioenergetic phenotype of VSMCs, which become more oxidative and less glycolytic than under control conditions. These findings point towards higher energy demand during β-glycerophosphate-induced transdifferentiation that is, probably, required to fuel cellular activity during this VSMC phenotype switch [17–20, 24, 25]. VSMCs may, thus, respond to the new energy demand by increasing ATP generation via mitochondrial oxidative phosphorylation, due to its superior efficiency [26, 36]. Of note, ATP is also used by the ectonucleotide pyrophosphatase/phosphodiesterase 1 (ENPP1) to generate the essential calcification inhibitor pyrophosphate [40], which may show a compensatory increase after phosphate exposure [41]. Previous studies described a decrease in total ATP content in VSMCs during osteo-/chondrogenic transdifferentiation [20]. However, the levels of cellular ATP do not safely reflect mitochondrial or glycolytic function [42]. This measurement reflects the sum of ATP-producing and ATP-consuming processes and not the changes in ATP generation, while the ATP production rates provide a better understanding of the function of the energy-producing pathways. We observed no change of total ATP levels in VSMCs following 24 h β-glycerophosphate exposure.

Moreover, the mitochondrial fuel usage is modified in VSMCs in response to β-glycerophosphate exposure. These cells have a higher ability to increase mitochondrial glutamine and long-chain fatty acid usage as substrates for oxidation, but not glucose oxidation. Mitochondria are able to adapt by changing their dependency and flexibility for specific oxidation substrates, to maintain the energy supply [43]. The transition in fuel choice allows for maintenance of energy and glucose homeostasis [44]. In accordance with these observations, a study shows that mitochondrial glucose oxidation is reduced while fatty acid oxidation is increased during transdifferentiation of VSMCs [25]. In β-glycerophosphate-treated VSMCs, the use of glucose and its metabolites may, thus, be redirected to other pathways involved in biosynthesis [17], similar as described in cancer cells [35, 36].

In addition to the bioenergetic function [19, 26, 39], mitochondria are also a source of reactive oxygen species (ROS) as a by-product of ATP production [39]. While low levels of ROS act as intracellular signalling molecules [45–47], high ROS levels induce oxidative stress and oxidative damage [46]. In accordance, the present observations describe that exposure to β-glycerophosphate promotes oxidative stress in VSMCs, effects blunted by inhibition of complex I and complex III and, thus, of mitochondrial respiration. The major source of mitochondrial ROS is represented by the complex I of the electron transport chain [46], but much lower levels of ROS can also be produced by the complex III and other mitochondrial enzymes [46, 47]. The generation of ROS by complex I is increased when the coupling efficiency of mitochondrial respiration to ATP production is reduced and the proton motive force is increased [46, 47] as well as when the NADH/NAD^+^ ratio is high [46]. We show here that β-glycerophosphate-treated VSMCs have an increased mitochondrial proton leak, leading to decreased coupling efficiency of mitochondrial respiration to mitochondrial ATP production, which may drive, at least in part, the β-glycerophosphate-induced oxidative stress in VSMCs [20, 29, 31]. The proton leak results on the one hand from a passive process and on the other hand from a process tightly regulated by the activity of several mitochondrial proteins [27, 47]. However, the mechanisms controlling proton leak are complex and poorly understood so far [27, 47]. Further studies are required to elucidate the underlying mechanisms of the β-glycerophosphate-induced proton leak and decrease in coupling efficiency in VSMCs.

In VSMCs, increased ROS levels may induce mitochondrial damage leading to mitochondrial dysfunction [17]. Despite the low contribution of mitochondrial oxidative phosphorylation to total ATP production in VSMCs, mitochondrial dysfunction has been associated with high phosphate and its detrimental consequences in VSMCs [5, 21–23, 29]. In addition to the oxidative stress, mitochondrial dysfunction may be induced by a multitude of other mechanisms [34, 39], which may also play a role during hyperphosphatemic conditions. Additional research is needed to elucidate the effects of phosphate on mitochondrial dysfunction in VSMCs and the mechanisms involved.

Mitochondrial oxidative phosphorylation and glycolytic reserve capacities are shown to be critical for vascular function [19], and failure to respond to the bioenergetic requirements leads to pathological processes [19, 34]. In general, cells maintain a high reserve capacity to accomplish physiological responses [34]. The bioenergetic reserve capacity is influenced by mitochondrial dysfunction and the activity of the components of the mitochondrial electron transport chain [34]. Even if β-glycerophosphate induces a more oxidative phenotype of VSMCs, the spare respiratory capacity is decreased, while the glycolytic reserve tends to be higher. Thus, the VSMCs may be able to respond to a further challenge with high energy demand by quick ATP supply especially via glycolysis. The respiratory reserve capacity is an indicator of mitochondrial health [34]. These findings are confirmed by the present data showing that β-glycerophosphate-treated VSMCs have a low metabolic potential for oxidative phosphorylation and, thus, a reduced ability to respond to stressed conditions through mitochondrial respiration.

In addition to the effects on mitochondrial function, the present study identified other metabolic changes induced by β-glycerophosphate in VSMCs. β-Glycerophosphate stimulated extracellular acidification resulting from other cellular processes than mitochondrial respiration and glycolysis [48]. The underlying mechanisms of this regulation and the potential role of the specific metabolic pathways in the detrimental effects of β-glycerophosphate require further study.

Taken together, the present study shows changes in cellular bioenergetics of VSMCs induced by exposure to elevated β-glycerophosphate levels. Hyperphosphatemia plays a critical role in the pathophysiology of vascular calcification in CKD [5, 6], a process which is mediated by and requires osteo-/chondrogenic transdifferentiation of VSMCs [5, 8]. A key role of VSMCs bioenergetic metabolism in vascular calcification is supported by previous studies [21–23, 29]. Moreover, we show here that targeting key metabolic components of mitochondrial respiration impacts on β-glycerophosphate-induced osteo-/chondrogenic transdifferentiation and calcification of VSMCs in vitro. In accordance with previous observations [31], inhibition of ATP synthase with oligomycin and, thus, mitochondrial ATP production does not affect, while blocking the mitochondrial respiration with rotenone and antimycin A suppresses β-glycerophosphate-induced osteo-/chondrogenic transdifferentiation and mineralization of VSMCs. Surprisingly, disrupting the mitochondrial membrane potential with the protonophore FCCP tends to augment β-glycerophosphate-induced osteo-/chondrogenic signalling in VSMCs, but does not impact mineralization of VSMCs. Even if the mitochondrial uncoupler should decrease the production of ROS [49], the potential extramitochondrial effects such as plasma membrane depolarization, cytotoxicity or apoptosis [49, 50] may drive osteoinduction and, thus, exacerbate osteo-/chondrogenic transdifferentiation in the presence of β-glycerophosphate [5, 20]. Of note, anti-calcific effects of another protonophore CCCP have been described in VSMCs [31]. The difference between the studies may be due to the cell types and the protonophore used. Thus, our data have revealed the mitochondrial respiratory chain as a key component and potential target at the onset of osteo-/chondrogenic signalling in VSMCs. Especially, the prevention of mitochondrial dysfunction may inhibit vascular calcification [21–23]. Mitochondrial therapeutics were already suggested for cardiovascular diseases [34, 39]. Moreover, modulation of mitochondrial proton leak is considered a potential therapeutic target in many diseases [47]. However, the specific metabolic pathways and the key components that are critical for phosphate-induced vascular calcification have to be further investigated. In-depth understanding of the metabolic pathways and bioenergetic phenotype of VSMCs during elevated phosphate conditions may advance the knowledge regarding the osteoinductive signalling leading to vascular calcification and provide targeted therapies to reduce the progression of vascular calcification in CKD. The current study is limited by a single early timepoint investigated and the use of β-glycerophosphate as phosphate donor, a widely used model for hyperphosphatemic calcification in cultured VSMCs [13, 15, 22, 31]. However, the current observations cannot rule out phosphate-independent effects of β-glycerophosphate on the bioenergetic profile. Furthermore, how bioenergetic alterations triggered by phosphate could affect the metabolism in CKD patients requires further study.

In conclusion, β-glycerophosphate modifies cellular bioenergetics in VSMCs by promoting a more oxidative phenotype, effects involving regulation of key parameters of mitochondrial function. Preventing the shift in the bioenergetic profile of VSMCs during hyperphosphatemic conditions may represent a therapeutic option to inhibit onset of osteo-/chondrogenic transdifferentiation of VSMCs and to reduce the progression of vascular calcification in CKD.

## Electronic supplementary material

ESM 1 (PDF 208 kb)
