# Supplementary material for: Impact of β-glycerophosphate on the bioenergetic profile of vascular smooth muscle cells
Source: J Mol Med (Berl). 2020 Jun 2;98(7):985–97. doi: 10.1007/s00109-020-01925-8 (PMC7343738; doi:10.1007/s00109-020-01925-8)

Impact of β-glycerophosphate on the bioenergetic profile of vascular smooth muscle cells

Ioana Alesutan[1,2,3,4], Franco Moritz[5], Tatjana Haider[1], Sun Shouxuan[1], Can Gollmann-Tepeköylü[6], Johannes Holfeld[6], Burkert Pieske[2,3,4,7], Florian Lang[8], Kai-Uwe Eckardt[9], Silke Sophie Heinzmann[5], Jakob Voelkl[1,2,4,9]

[1]Institute for Physiology and Pathophysiology, Johannes Kepler University, Linz, Austria; [2]DZHK (German Centre for Cardiovascular Research), partner site Berlin, Berlin, Germany; [3]Berlin Institute of Health (BIH), Berlin, Germany; [4]Department of Internal Medicine and Cardiology, Charité – Universitätsmedizin Berlin, Campus Virchow-Klinikum, Berlin, Germany; [5]Analytical BioGeoChemistry, Helmholtz Zentrum München, German Research Center for Environmental Health, Neuherberg, Germany; [6]University Clinic of Cardiac Surgery, Medical University of Innsbruck, Innsbruck, Austria; [7]Department of Internal Medicine and Cardiology, German Heart Center Berlin (DHZB), Berlin, Germany; [8]Department of Physiology I, Eberhard-Karls University, Tübingen, Germany; [9]Department of Nephrology and Medical Intensive Care, Charité – Universitätsmedizin Berlin, Campus Virchow-Klinikum, Berlin, Germany.

Supplemental material

Materials and methods

Cell culture of primary human aortic smooth muscle cells

Primary human aortic smooth muscle cells (HAoSMCs) were commercially obtained from Sigma Aldrich (Cat. 354-05A) [1] and cultured in growth medium containing a 1:1 ratio of Waymouth's MB 752/1 and Ham's F-12 nutrient mixture (containing ~1.85 mM phosphate according to manufacturer's information) supplemented with 10% FBS, 100 U/ml penicillin and 100 µg/ml streptomycin (Thermo Fisher Scientific) [2-4]. Each experiment was performed in at least two different batches of HAoSMCs at different passages (from 4 to 11), depending on the availability of the cells. At confluence, HAoSMCs were split into 6-well plates ($2x10^5$ cells/well), 96-well plates ($6x10^3$ cells/well) or Seahorse 96-well XF microplates ($6x10^3$ cells/well; Agilent Technologies) and allowed to attach for 24 hours prior to treatment for 24 hours with 2 mM β-glycerophosphate (Sigma Aldrich), 1 µM Oligomycin (Agilent Technologies), 1 µM each of rotenone and antimycin A mix (Agilent Technologies) or 0.75 µM carbonyl cyanide-4 (trifluoromethoxy) phenylhydrazone (FCCP, Agilent Technologies). Treatment for 11 days with calcification medium containing 10 mM β-glycerophosphate and 1.5 mM $CaCl_2$ (Sigma-Aldrich) was used for the calcification assay [2, 5-7]. Fresh media with agents were added every 2-3 days.

Extracellular flux analysis

The Seahorse XFe96 Analyzer (Agilent Biotechnologies) was used to simultaneously monitor mitochondrial respiration and glycolysis in HAoSMCs by determining the oxygen consumption rate (OCR) and extracellular acidification rate (ECAR), respectively. The Seahorse XF Cell Mito Stress Test kit, Seahorse XF Mito Fuel Flex Test kit, Seahorse XF Glycolysis Stress Test kit, Seahorse XF Glycolytic Rate Assay kit, Seahorse XF Real-Time ATP Rate Assay kit and Seahorse XF Cell Energy Phenotype Test kit (all from Agilent Biotechnologies) were used according to the manufacturer's protocols.

The day prior to assays, sensor cartridges were hydrated in Seahorse XF Calibrant solution (Agilent Biotechnologies) by incubation overnight at 37°C in a non-CO_2 incubator. The medium of HAoSMCs treated in the Seahorse 96-well XF microplates as described above, was replaced with assay medium one hour prior to assays and HAoSMCs were incubated at 37°C in a non-CO_2 incubator until the measurements. The assay medium used for the measurements was Seahorse XF DMEM medium, pH 7.4 (Agilent Biotechnologies) supplemented with 1 mM pyruvate (Agilent Biotechnologies), 2 mM glutamine (Agilent Biotechnologies) and 19 mM glucose (Agilent Biotechnologies). For the Glycolysis Stress

Test, assay medium without pyruvate and glucose supplementation was used. During the assays, 1 µM Oligomycin (Oligo), 0.75 µM FCCP, a mix of 1 µM each rotenone and antimycin A (Rot/AA), 19 mM glucose, 50 mM 2-deoxy-glucose (2-DG), 3 µM BPTES, 160 µM Etomoxir and/or 80 µM UK5099 were simultaneously/sequentially injected as indicated in the figures. At the end of the assays, HAoSMCs were lysed in IP lysis buffer (Thermo Fisher Scientific) and protein concentration was determined by Bradford assay (Bio-Rad Laboratories).

The readings were normalized to total protein concentration of each well. The Seahorse assays were performed at least in duplicate and the average of the results was used. The proton efflux rates (PER) and glyco-PER were determined by converting automatically the experimentally-derived OCR and ECAR data using the Glycolytic Rate Assay Report Generator of Seahorse Wave Desktop software (Agilent Biotechnologies), according to the manufacturer's protocol. Due to differences in mitochondrial respiration and glycolysis during passaging of HAoSMCs (data not shown) [8], the OCR, ECAR and PER rates of each experiment were normalized to the value of the first measurement in the control group during the time course of the assay. ATP production rates were normalized to protein content only.

Parameters of mitochondrial function were calculated from Cell Mito Stress Test, according to the manufacturer's protocol. Non-mitochondrial OCR was calculated as OCR after rotenone/antimycin A injection. Basal respiration was calculated as the difference between the OCR before first injection and non-mitochondrial OCR. ATP production was calculated as the difference between basal and oligomycin-inhibited OCR. Maximal respiration was calculated as the difference between FCCP-induced OCR and non-mitochondrial respiration. Spare respiratory capacity was calculated as the difference between maximal and basal respiration. Spare respiratory capacity as percentage was calculated as the ratio between maximal and basal respiration multiplied by 100. Proton leak was calculated as the difference between oligomycin-inhibited OCR and non-mitochondrial OCR. The coupling efficiency as percentage was calculated as the ratio between ATP production and basal respiration multiplied by 100.

Parameters of mitochondrial fuel usage were calculated from Mito Fuel Flex Test, according to the manufacturer's protocol. Dependency as percentage was calculated as the ratio between the difference of basal OCR and OCR after target pathway inhibition and the difference of basal OCR and OCR after alternative two pathways inhibition, multiplied by 100. Capacity as percentage was calculated as the difference between 1 and the ratio between the difference of basal OCR and OCR after alternative two pathways inhibition and the difference

of basal OCR and OCR after target pathway inhibition, multiplied by 100. Flexibility was calculated as the difference between capacity and dependency.

Parameters of glycolytic function were calculated from Glycolysis Stress Test, according to the manufacturer's protocol. Non-glycolytic acidification was calculated as ECAR before glucose injection. Glycolysis was calculated as the difference between glucose-induced ECAR and non-glycolytic acidification. Glycolytic capacity was calculated as the difference between oligomycin-induced ECAR and non-glycolytic acidification. Glycolytic reserve was calculated as the difference between glycolytic capacity and glycolysis. Glycolytic reserve as percentage was calculated as the ratio between glycolytic capacity and glycolysis, multiplied by 100.

Parameters of glycolytic rates were calculated from Glycolytic Rate Assay, according to the manufacturer's protocol. Basal Glycolysis was calculated as glyco-PER before rotenone/antimycin A injection. Basal PER was calculated as PER before rotenone/antimycin A injection. PER from glycolysis as percentage was calculated as the ratio between basal glycolysis and basal PER, multiplied by 100. Compensatory glycolysis was calculated as rotenone/antimycin A-induced glyco-PER. Post-2-DG acidification was calculated as PER after 2-DG injection.

ATP production rates were calculated from Real-Time ATP Rate Assay, according to the manufacturer's protocol. Mito-ATP production rates were calculated as the difference between OCR before and after oligomycin injection. Glyco-ATP production rates were calculated as glyco-PER before oligomycin injection. Total ATP production rates were calculated as the sum of mito-ATP and glyco-ATP production rates. ATP rate index was calculated as the ratio between mito-ATP and glyco-ATP production rates.

Parameters of cell energy phenotype were calculated from Cell Energy Phenotype Test, according to the manufacturer's protocol. Baseline OCR/ECAR was calculated as the measurement before oligomycin/FCCP injection. Stressed OCR/ECAR was calculated as the measurement after oligomycin/FCCP injection. Metabolic potential OCR/ECAR as percentage was calculated as the ratio between stressed OCR/ECAR and baseline OCR/ECAR, multiplied by 100.

RNA isolation and quantitative RT-PCR

Total RNA was isolated from HAoSMCs by using Trizol Reagent (Thermo Fisher Scientific) [9-11]. cDNA was synthesized by using oligo(dT)$_{12\text{-}18}$ primers (Thermo Fisher Scientific) and SuperScript III Reverse Transcriptase (Thermo Fisher Scientific). Quantitative

RT-PCR was performed in duplicate with iQ Sybr Green Supermix (Bio-Rad Laboratories) and CFX96 Real-Time PCR Detection System (Bio-Rad Laboratories). The following human primers were used (Thermo Fisher Scientific, 5’→3’ orientation) [3, 5, 6, 12]:

ALPL fw: GGGACTGGTACTCAGACAACG;

ALPL rev: GTAGGCGATGTCCTTACAGCC;

BMP2 fw: TTCGGCCTGAAACAGAGACC;

BMP2 rev: CCTGAGTGCCTGCGATACAG;

CBFA1 fw: GCCTTCCACTCTCAGTAAGAAGA;

CBFA1 rev: GCCTGGGGTCTGAAAAAGGG;

GAPDH fw: GAGTCAACGGATTTGGTCGT;

GAPDH rev: GACAAGCTTCCCGTTCTCAG;

OPN fw: GAAGTTTCGCAGACCTGACAT;

OPN rev: GTATGCACCATTCAACTCCTCG;

SP7 fw: CACAAAGAAGCCGTACTCTGT;

SP7 rev: GGGGCTGGATAAGCATCCC;

TAGLN fw: CCGTGGAGATCCCAACTGG;

TAGLN rev: CCATCTGAAGGCCAATGACAT.

The specificity of the PCR products was confirmed by analysis of the melting curves and relative mRNA expression was calculated by the $2^{-\Delta\Delta Ct}$ method using GAPDH as housekeeping gene, normalized to the control group.

Genomic DNA isolation and mtDNA copy number analysis

Genomic DNA was isolated from HAoSMCs 24 hours following treatments by using the NucleoSpin Tissue kit (Macherey-Nagel) according to the manufacturer’s protocol. Mitochondrial DNA (mtDNA) copy number was determined relative to nuclear DNA copy number by quantitative RT-PCR [13, 14] with CFX96 Real-Time PCR Detection System (Bio-Rad Laboratories). RT-PCR was performed in duplicate by using 20 ng’s genomic DNA, iQ Sybr Green Supermix (Bio-Rad Laboratories) and the following human primers (Thermo Fisher Scientific; [14]):

Mitochondrial:

mt3211 fw: CACCCAAGAACAGGGTTTGT;

mt3211 rev: TGGCCATGGGTATGTTGTTAA;

mt9827 fw: CGTCATTATTGGCTCAAC;

mt9827 rev: GATGGAGACATACAGAAATAG;

Nuclear:

B2M fw: TGCTGTCTCCATGTTTGATGTATCT;

B2M rev: TCTCTGCTCCCCACCTCTAAGT.

The specificity of the PCR products was confirmed by analysis of the melting curves and relative mtDNA expression was calculated by the $2^{-\Delta\Delta Ct}$ method for two mitochondrial DNA segments and the average of these results was used, normalized to the control group.

Total antioxidant capacity assay

Total antioxidant capacity was measured in cell lysate by using the colorimetric antioxidant assay kit (Cayman Chemical) according to the manufacturer's protocol [6, 15]. To calculate the relative total antioxidant capacity Trolox was used as standard. The results were normalized to total protein concentration measured by the Bradford assay (Bio-Rad Laboratories) and to the control group.

H_2O_2 levels

H_2O_2 levels were measured in cell lysate by using the colorimetric hydrogen peroxide assay kit (Abcam) according to the manufacturer's protocol. Total protein concentration was measured by the Bradford assay (Bio-Rad Laboratories). Prior to assay, samples were deproteinized in 1 M PCA solution. PCA was subsequently neutralized and removed from supernatant by precipitation with 2 M KOH. The results were normalized to total protein concentration and to the control group.

Lactate levels

Lactate levels were measured in cell culture medium from HAoSMCs by using the colorimetric lactate assay kit (Sigma Aldrich) according to the manufacturer's protocol. Prior to assay, samples were deproteinized in 1 M PCA solution. PCA was subsequently neutralized and removed from supernatant by precipitation with 2 M KOH. HAoSMCs were lysed in IP lysis buffer (Thermo Fisher Scientific) and total protein concentration was measured by the Bradford assay (Bio-Rad Laboratories). The results were normalized to total protein concentration and to the control group.

ATP levels

ATP levels in HAoSMCs were measured by using the luminescent ATP detection assay kit (Abcam) and a microplate luminometer (GloMax Navigator System, Promega) according to the manufacturer's protocol. The results were normalized to the control group.

ATP synthase activity assay

HAoSMCs were lysed and ATP synthase activity was measured by using the colorimetric ATP synthase enzyme activity assay kit (Abcam) according to the manufacturer's protocol. Total protein concentration was measured by the Bradford assay (Bio-Rad Laboratories). The results were normalized to total protein concentration and to the control group.

Calcification analysis

To quantify mineralization, HAoSMCs were incubated in 0.6 M HCl for 24 hours at 4°C. The calcium content in the supernatant was determined by using QuantiChrom Calcium assay kit (BioAssay Systems) according to the manufacturer's protocol. HAoSMCs were lysed with 0.1 M NaOH/0.1% SDS and total protein concentration was determined by the Bradford assay (Bio-Rad Laboratories). Results are shown normalized to total protein concentrations [2, 5, 6].

Statistical analysis

Data are shown as scatter dot plots and arithmetic means ± SEM and *n* indicates the number of independent experiments performed at different passages of the cells [9, 15]. Normality was tested with Shapiro-Wilk test. Non-normal datasets were transformed (log, sqrt or reciprocal) prior to statistical testing to provide normality. Two groups were compared by unpaired two-tailed t-test or Mann-Whitney u-test. Statistical testing of more than two groups was performed by one-way ANOVA followed by Tukey's HSD test (homoscedastic data) or Games-Howell test (heteroscedastic data). $P<0.05$ was considered statistically significant.

References

1. Luong TTD, Estepa M, Boehme B, Pieske B, Lang F, Eckardt KU, Voelkl J, Alesutan I (2019) Inhibition of vascular smooth muscle cell calcification by vasorin through interference with TGFbeta1 signaling. Cell Signal: 109414. DOI 10.1016/j.cellsig.2019.109414
2. Voelkl J, Tuffaha R, Luong TTD, Zickler D, Masyout J, Feger M, Verheyen N, Blaschke F, Kuro OM, Tomaschitz A, et al. (2018) Zinc Inhibits Phosphate-Induced Vascular Calcification through TNFAIP3-Mediated Suppression of NF-kappaB. J Am Soc Nephrol 29: 1636-1648. DOI 10.1681/ASN.2017050492
3. Voelkl J, Alesutan I, Leibrock CB, Quintanilla-Martinez L, Kuhn V, Feger M, Mia S, Ahmed MS, Rosenblatt KP, Kuro OM, et al. (2013) Spironolactone ameliorates PIT1-dependent vascular osteoinduction in klotho-hypomorphic mice. J Clin Invest 123: 812-822. DOI 10.1172/JCI64093
4. Alesutan I, Tuffaha R, Auer T, Feger M, Pieske B, Lang F, Voelkl J (2017) Inhibition of osteo/chondrogenic transformation of vascular smooth muscle cells by MgCl2 via calcium-sensing receptor. J Hypertens 35: 523-532. DOI 10.1097/HJH.0000000000001202
5. Henze LA, Luong TTD, Boehme B, Masyout J, Schneider MP, Brachs S, Lang F, Pieske B, Pasch A, Eckardt KU, et al. (2019) Impact of C-reactive protein on osteo-/chondrogenic transdifferentiation and calcification of vascular smooth muscle cells. Aging (Albany NY) 11: 5445-5462. DOI 10.18632/aging.102130
6. Boehme B, Schelski N, Makridakis M, Henze L, Vlahou A, Lang F, Pieske B, Alesutan I, Voelkl J (2018) Role of Cytosolic Serine Hydroxymethyl Transferase 1 (SHMT1) in Phosphate-Induced Vascular Smooth Muscle Cell Calcification. Kidney Blood Press Res 43: 1212-1221. DOI 10.1159/000492248
7. Villa-Bellosta R, Millan A, Sorribas V (2011) Role of calcium-phosphate deposition in vascular smooth muscle cell calcification. Am J Physiol Cell Physiol 300: C210-220. DOI 10.1152/ajpcell.00229.2010
8. Yang M, Chadwick AE, Dart C, Kamishima T, Quayle JM (2017) Bioenergetic profile of human coronary artery smooth muscle cells and effect of metabolic intervention. PLoS One 12: e0177951. DOI 10.1371/journal.pone.0177951
9. Voelkl J, Luong TT, Tuffaha R, Musculus K, Auer T, Lian X, Daniel C, Zickler D, Boehme B, Sacherer M, et al. (2018) SGK1 induces vascular smooth muscle cell calcification through NF-kappaB signaling. J Clin Invest 128: 3024-3040. DOI 10.1172/JCI96477
10. Schelski N, Luong TTD, Lang F, Pieske B, Voelkl J, Alesutan I (2019) SGK1-dependent stimulation of vascular smooth muscle cell osteo-/chondrogenic transdifferentiation by interleukin-18. Pflugers Arch 471: 889-899. DOI 10.1007/s00424-019-02256-5
11. Luong TTD, Schelski N, Boehme B, Makridakis M, Vlahou A, Lang F, Pieske B, Alesutan I, Voelkl J (2018) Fibulin-3 Attenuates Phosphate-Induced Vascular Smooth Muscle Cell Calcification by Inhibition of Oxidative Stress. Cell Physiol Biochem 46: 1305-1316. DOI 10.1159/000489144
12. Wang X, Seed B (2003) A PCR primer bank for quantitative gene expression analysis. Nucleic Acids Res 31: e154. DOI 10.1093/nar/gng154
13. Yu EPK, Reinhold J, Yu H, Starks L, Uryga AK, Foote K, Finigan A, Figg N, Pung YF, Logan A, et al. (2017) Mitochondrial Respiration Is Reduced in Atherosclerosis, Promoting Necrotic Core Formation and Reducing Relative Fibrous Cap Thickness. Arterioscler Thromb Vasc Biol 37: 2322-2332. DOI 10.1161/ATVBAHA.117.310042
14. Gammage PA, Van Haute L, Minczuk M (2016) Engineered mtZFNs for Manipulation of Human Mitochondrial DNA Heteroplasmy. Methods Mol Biol 1351: 145-162. DOI 10.1007/978-1-4939-3040-1_11
15. Alesutan I, Feger M, Tuffaha R, Castor T, Musculus K, Buehling SS, Heine CL, Kuro OM, Pieske B, Schmidt K, et al. (2016) Augmentation of phosphate-induced osteo-/chondrogenic transformation of vascular smooth muscle cells by homoarginine. Cardiovasc Res 110: 408-418. DOI 10.1093/cvr/cvw062

Supplemental Figures

Suppl. Fig. 1. Effect of β-glycerophosphate on mitochondrial glucose usage in primary human aortic smooth muscle cells. Arithmetic means ± SEM (n=5; arbitrary units, a.u.) of normalized oxygen consumption rate (OCR) measured over time in HAoSMCs following treatment for 24 hours with control (CTR) or β-glycerophosphate (β-GP). Glucose dependency (**a**) and capacity (**b**) were determined by sequential injection of UK5099 and a mix of BPTES and Etomoxir (Eto) inhibitors at the indicated times.

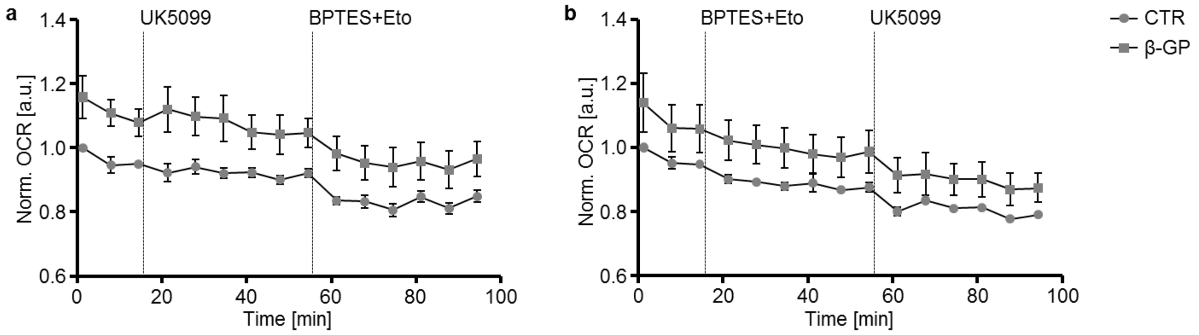

Suppl. Fig. 2. Effect of β-glycerophosphate on mitochondrial glutamine usage in primary human aortic smooth muscle cells. Arithmetic means ± SEM (n=5; arbitrary units, a.u.) of normalized oxygen consumption rate (OCR) measured over time in HAoSMCs following treatment for 24 hours with control (CTR) or β-glycerophosphate (β-GP). Glutamine dependency (**a**) and capacity (**b**) were determined by sequential injection of BPTES and a mix of UK5099 and Etomoxir (Eto) inhibitors at the indicated times.

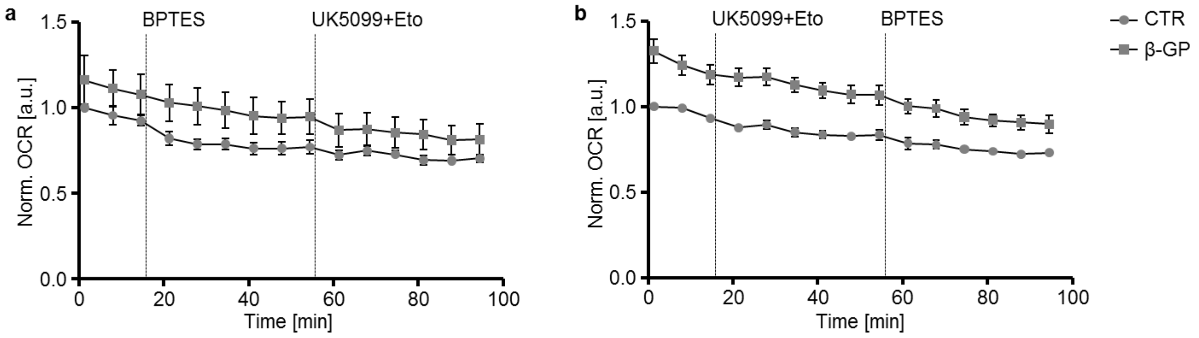

Suppl. Fig. 3. Effect of β-glycerophosphate on mitochondrial fatty acid usage in primary human aortic smooth muscle cells. Arithmetic means ± SEM (n=5; arbitrary units, a.u.) of normalized oxygen consumption rate (OCR) measured over time in HAoSMCs following treatment for 24 hours with control (CTR) or β-glycerophosphate (β-GP). Fatty acid dependency (**a**) and capacity (**b**) were determined by sequential injection of Etomoxir (Eto) and a mix of BPTES and UK5099 inhibitors at the indicated times.

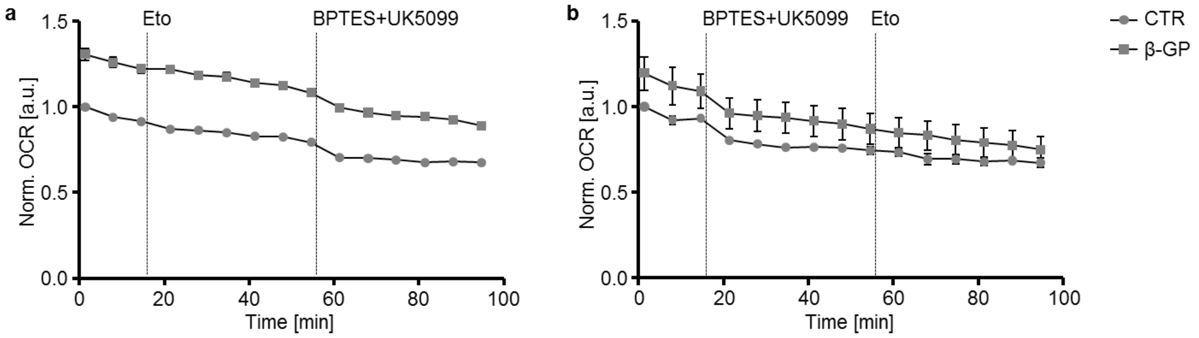

Supplement: Supplementary file 1 — (PDF 208 kb) [file 109_2020_1925_MOESM1_ESM.pdf]
